# Effect of D-amino acid metabolic enzyme deficiency on cancer development—diffuse large B-cell lymphoma onset and gene expression analyses in *DASPO*-knockout mice

**DOI:** 10.1007/s00726-024-03426-1

**Published:** 2024-12-24

**Authors:** Yusuke Nakade, Yasunori Iwata, Kenichi Harada, Yasuharu Sato, Masashi Mita, Kenji Hamase, Ryuichi Konno, Mayo Hayashi, Taku Kobayashi, Yuta Yamamura, Tadashi Toyama, Atsushi Tajima, Takashi Wada

**Affiliations:** 1 Department of Nephrology and Rheumatology, Kanazawa University, 13-1 Takara-Machi, Kanazawa, 920-8641 Japan; 2 Department of Clinical Laboratory, Kanazawa University Hospital, 13-1 Takara-Machi, Kanazawa, 920-8641 Japan; 3 Department of Human Pathology, Kanazawa University Graduate School of Medicine, 13-1 Takara-Machi, Kanazawa, 920-8641 Japan; 4 Department of Molecular Hematopathology, Okayama University Graduate School of Health Sciences, 2-5-1 Shikata-Chou, Kita-Ku, Okayama, 700-8558 Japan; 5 KAGAMI Co., Ltd., 7-18 Saitobaiohiruzu Centre 308, Ibaragi-Shi, Osaka, 567-0085 Japan; 6 Graduate School of Pharmaceutical Sciences, Kyushu University, 3-1-1 Maidashi, Higashi-Ku, Fukuoka, 812-8582 Japan; 7 Department of Pharmaceutical Sciences, International University of Health and Welfare, 2600-1 Kitakanemaru, Ohtawara, 324-8501 Japan; 8 Department of Bioinformatics and Genomics, Graduate School of Advanced Preventive Medical Sciences, Kanazawa University, 13-1 Takara-Machi, Kanazawa, 920-8641 Japan

**Keywords:** D-amino acid, D-amino acid oxidase, D-aspartate oxidase, Diffuse large B-cell lymphoma, Serine racemase

## Abstract

The relationship between D-AA metabolic enzymes and cancer development remains unclear. We aimed to investigate this relationship using mice deficient in D-AA-related metabolic enzymes. We examined mice lacking these enzymes for approximately 900 days and the effects of altered D-AA metabolism on cancer development based on lifespan, pathological findings, and gene expression. The lifespan of female *DASPO* -knockout (*DASPO*^*−/−*^) mice was shorter than that of the other group mice; furthermore, these mice showed tumor-like masses in the liver, spleen, and small intestine. A pathological diagnosis of diffuse large B-cell lymphoma (DLBCL) was made. RNA sequencing of the liver samples showed specific alterations in the expression of 71 genes in *DASPO*^*−/−*^ mice compared with that in wild-type B6 mice; *RGS 1*, *MTSS1*, and *SMARCD 1* were identified as DLBCL-related genes. Patients with DLBCL exhibiting low *DASPO* expression demonstrated a shorter survival period than those showing high expression. However, the role of *DASPO* in DLBCL development is unclear. Therefore, future research should focus on B cells. *DASPO* may serve as novel biomarkers and therapeutic targets in cancer.

**Graphical abstract:**

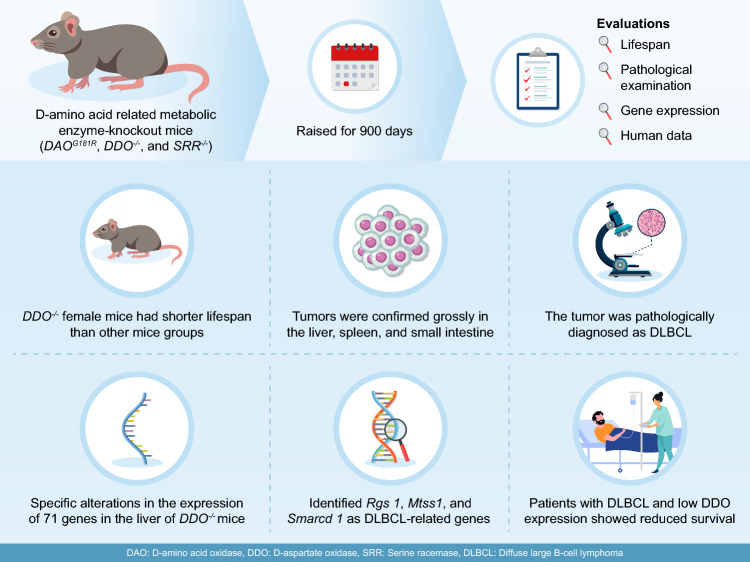

**Supplementary Information:**

The online version contains supplementary material available at 10.1007/s00726-024-03426-1.

## Introduction

For many years, only L-type amino acids were thought to exist in the human body and have biologically active functions. Chiral amino acid metabolomics (Miyoshi et al. 2012a, b) has revealed the existence and bioactive effects of D-amino acids (D-AAs) in humans (Iwata et al. 2022; Kimura et al. 2016; Nakade et al. 2018). D-Amino acid oxidase (DAAO) and D-aspartate oxidase (DASPO) are degrading enzymes that regulate D-AA levels (Ohide et al. 2011). DAAO uses neutral and basic D-AAs as substrates, whereas DASPO uses acidic D-AAs (D-glutamic acid and D-aspartic acid) as substrates and converts them to α-keto acids through oxidative deamination. Serine racemase (SR) is another D-AA-related enzyme that catalyzes the racemization of l-serine to d-serine (Wolosker et al. 1999).

The D-AA levels in the body are mainly regulated by D-AA-related metabolic enzymes and microbiota (Iwata et al. 2022; Nakade et al. 2018) under normal conditions. DAAO and SR activities are altered during renal failure (Nakade et al. 2018). Thus, D-AA-related metabolic enzymes may be involved in disease onset and progression. However, the congenital defects of these enzymes themselves and the long-term consequences of the abnormal D-AA metabolism caused by the defects remain unknown. The relationship between D-AAs and cancer development has been gaining attention (Bastings et al. 2019). Cancer cells utilize D-AAs by altering their metabolism for efficient cell growth (Pavlova and Thompson 2016). Therefore, we aimed to investigate the relationship between D-AA-related metabolic enzymes and cancer development using mice deficient in D-AA-related metabolic enzymes.

## Materials and methods

### Mice

**Fig. 1:**
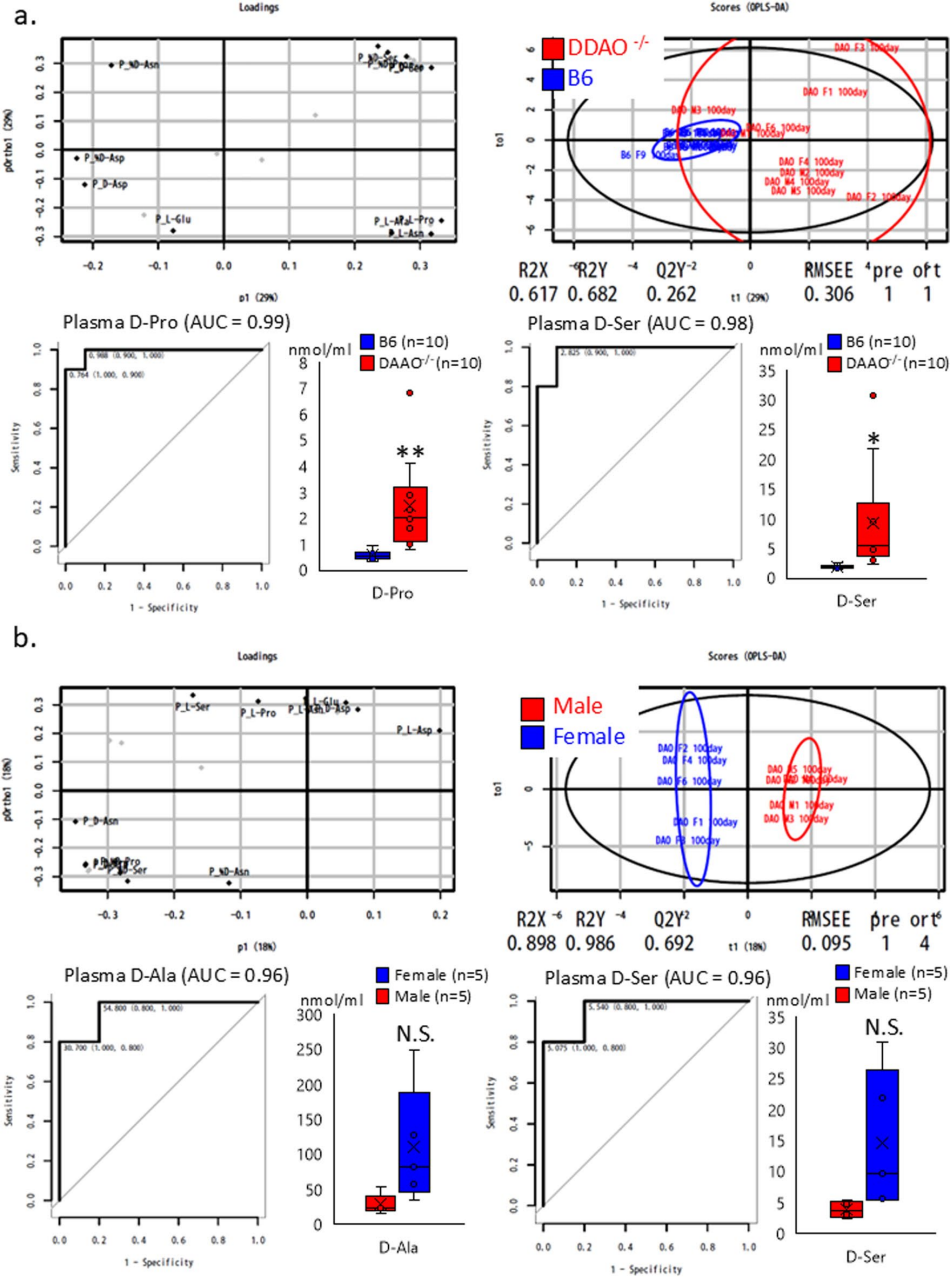

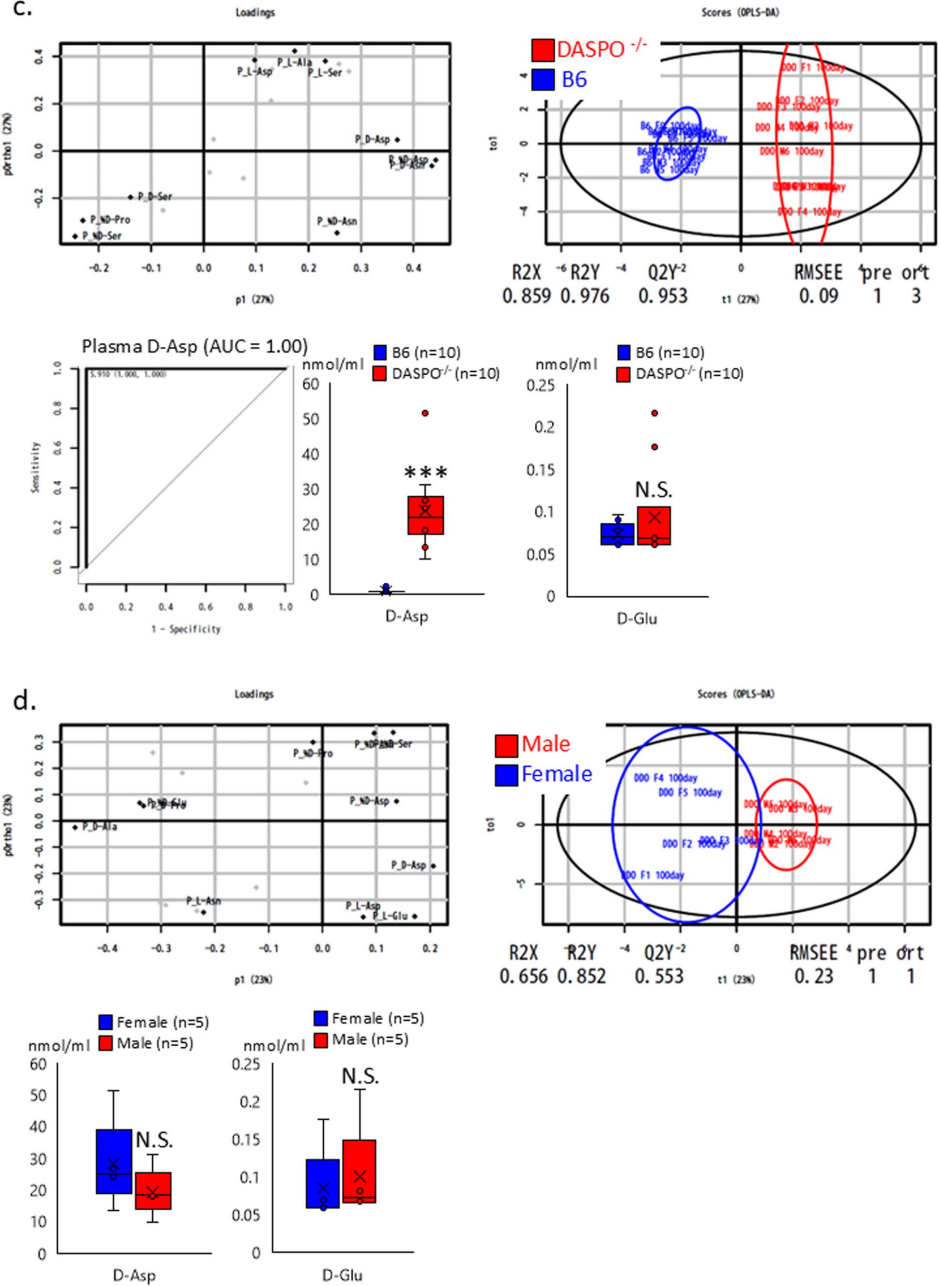

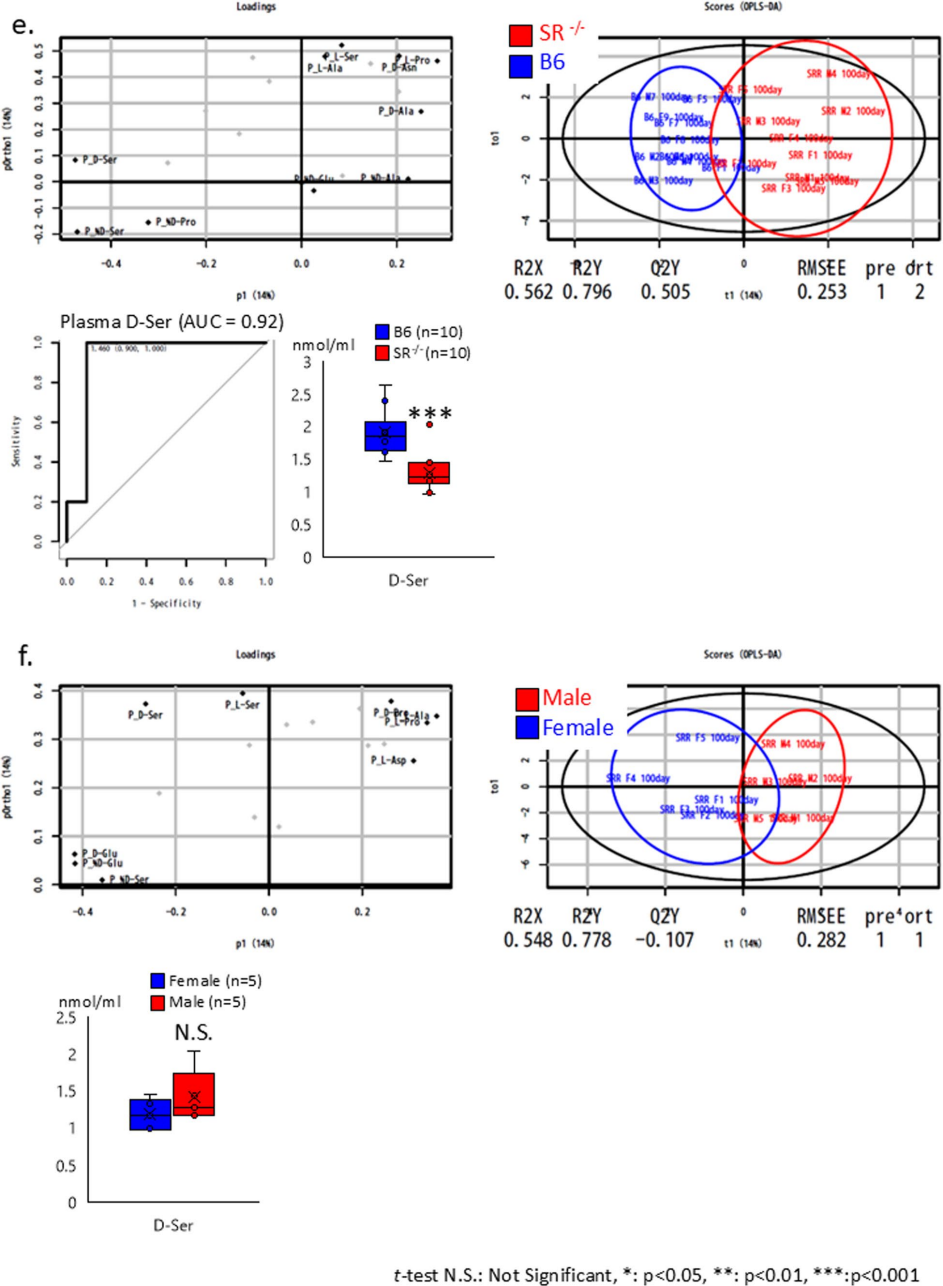
Plasma amino acid profiles. **a**, **c**, **e** Plasma D-AA concentrations in B6 and gene-deficient mice. **b**, **d**, **f** Comparisons between male and female mice. The concentrations of serine, alanine, asparagine, and proline, the major substrates of DAAO; serine, a substrate of SR; glutamic acid and aspartic acid, substrates of DASPO, were measured. D-AA, D-amino acid; DAAO, D-amino acid oxidase; DASPO, D-aspartate oxidase; SR, serine racemase; D-Ser, D-serine; D-Ala, D-alanine; D-Asn, D-asparagine; D-Pro, D-proline; D-Glu, D-glutamic acid; D-Asp; D-aspartic acid

**Fig. 2:**
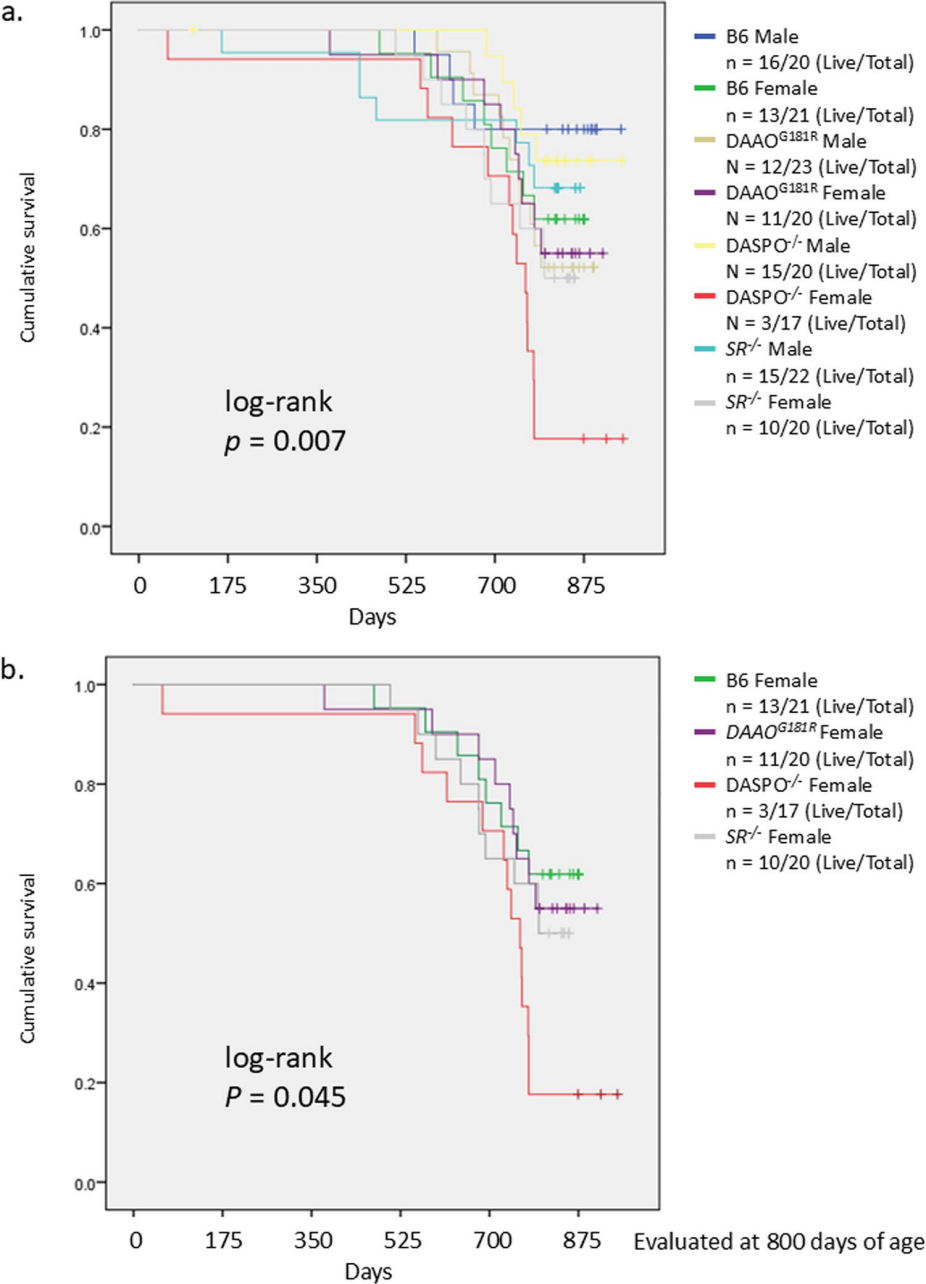
Survival curve. Survival rates were calculated at 800 days of age. Survival curve of **a** all B6 and D-AA-related metabolic enzyme-knockout mice (*DAAO*^*G181R*^, *DASPO*^*−/−*^, and *SR*^*−/*−^) and **b** all female B6 and D-AA-related metabolic enzyme-knockout mice. A log-rank test was performed for survival time analysis. D-AA, D-amino acid; DAAO, D-amino acid oxidase; DASPO, D-aspartate oxidase; SR, serine racemase

**Fig. 3:**
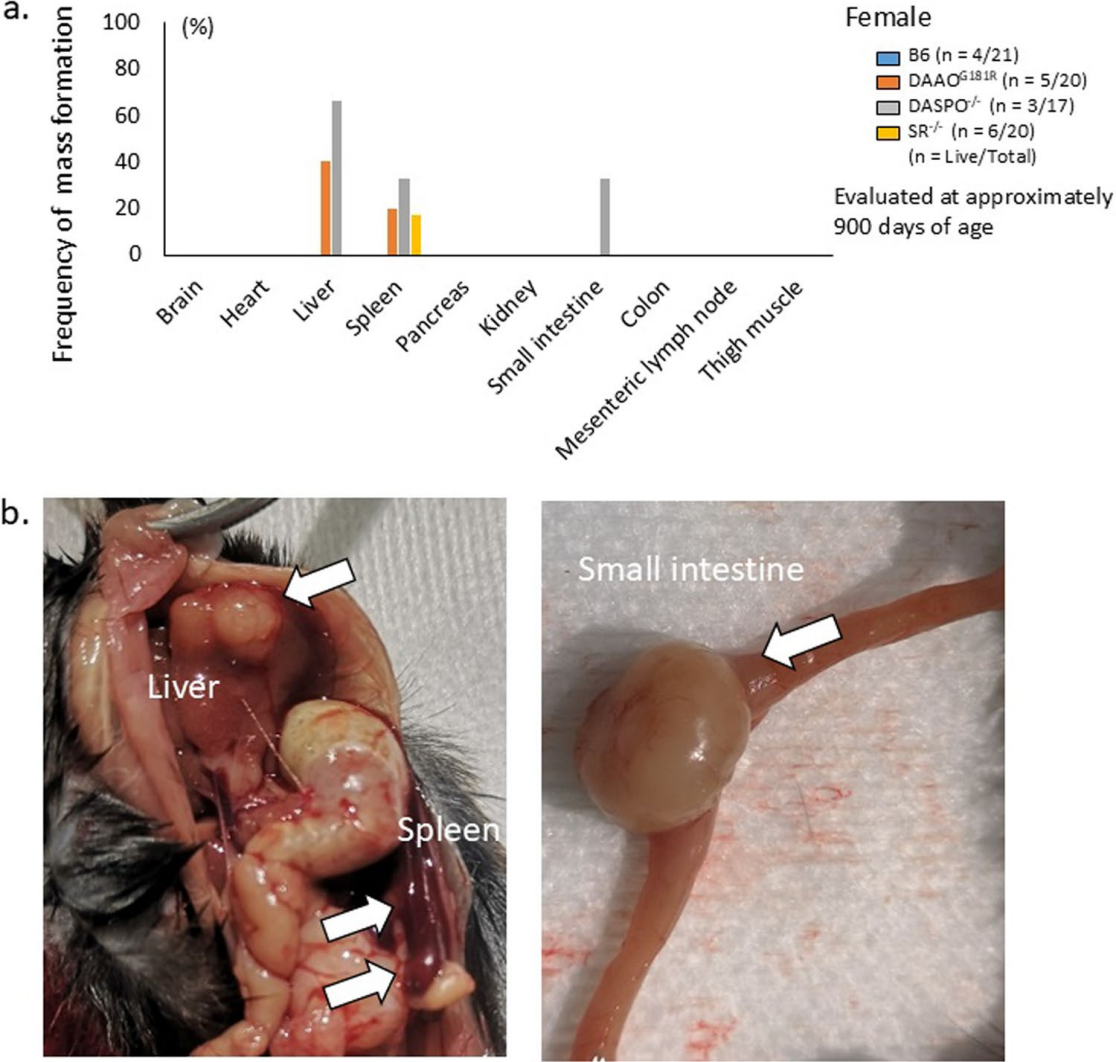
Macroscopic findings of organs in female mice. Macroscopic findings of organs were evaluated to determine the cause of death in female *DASPO*^*−/−*^ mice. The mice were sacrificed at approximately 900 days of age. **a** Frequency of mass formation in female B6 and D-AA-related metabolic enzyme-knockout mice (*DAAO*^*G181R*^, *DASPO*^*−/−*^, and *SR*^*−/−*^). **b** Macroscopic findings of organs in female *DASPO*^*−/−*^ mice. D-AA, D-amino acid; DAAO, D-amino acid oxidase; DASPO, D-aspartate oxidase; SR, serine racemase

**Fig. 4:**
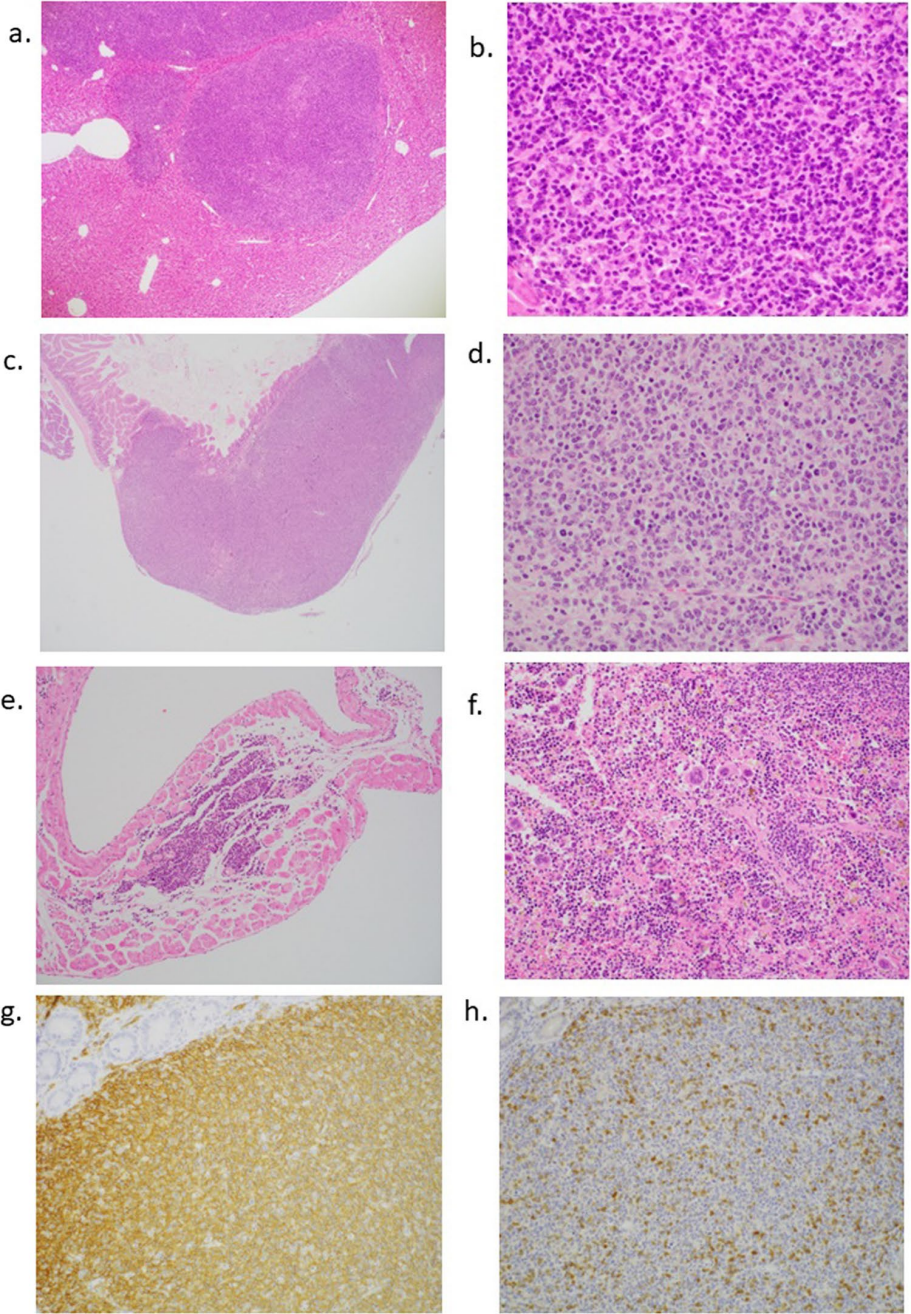
Pathological findings of each organ in female *DASPO*^*−/−*^ mice. **a** The liver showing multiple nodular infiltrations of lymphocytes. **b** A higher-magnification image of the liver. A mass consisting of medium to large atypical lymphocytes can be observed. **c** Small intestine showing cellular thickening of the wall. **d** A higher magnification of the small intestine showing the accumulation of medium to large atypical lymphocytes. **e** The atrial septum shows monotonous lymphocytes accumulated in the impulse-conducting system of the heart. **f** The spleen shows several multinucleated megakaryocytes. **g** Immunohistochemistry for CD20. Atypical lymphocytes were positive for CD20. **h** Immunohistochemistry for CD3. CD3-positive cells were scattered but among normal lymphocytes. Original magnification ×40 (**a**, **c**), ×100 (**e**), ×200 (**b**, **d**, **f**, **g**, **h**). DASPO, D-aspartate oxidase

B6 mice (CLEA Japan, Osaka, Japan) were housed and bred at Kanazawa University. DAAO^G181R^, *DASPO*-knockout (*DASPO*^*−/−*^), and *SR*-knockout (*SR*^*−/−*^) mouse lines generated and maintained in a B6 background were utilized in the study. We have previously reported that these mice lack target genes and cannot metabolize the relevant amino acids (Miyoshi et al. 2012a, b; Tojo et al. 2009; Han et al. 2015). The plasma amino acid profiles of approximately 100-day-old mice are shown in Fig. 1. The mice (n = 20–23) were bred and evaluated for longevity compared with the lifespan of B6 mice (Fig. 2). The evaluation of lifespan was conducted at 800 days of age. The mice were euthanized via isoflurane inhalation at approximately 900 days of age, and their brain, heart, liver, spleen, pancreas, kidney, small intestine, colon, mesenteric lymph node, and thigh muscle were examined (Figs. 3 and 4 and Online Resources 1 and 2). The breeding period before euthanasia did not exceed 30 days. Some mice died naturally during the 900-day breeding period. Approximately 20 mice were used to assess the survival period in the context of long-term breeding. All mice used in the experiment were evaluated. Randomization was not employed as we focused on comparing the lifespan of each KO mouse with that of B6 mice. The number of mice that survived for 900 days and the number of mice that died naturally are presented in Fig. 2 (survival rate: number of live mice/total number of mice). Cancer was confirmed as the cause of death. To monitor the health of the mice, regular assessments, including recording of weight, examination of hair unruliness, measurement of blood pressure, and sampling of blood and urine, were performed. Evaluations were conducted every 100 days until day 900. The research staff involved in the animal experiment at the Kanazawa University Animal Experiment Facility have received basic training.

To clarify the expression changes in genes involved in tumor (diffuse large B-cell lymphoma [DLBCL]) formation, we used the liver tissues of mice at 500 days of age (before disease onset), which was estimated to be just before tumor (DLBCL) formation based on the findings of long-term rearing (Fig. 2). To evaluate gene expression over time, the liver tissues from both B6 and *DASPO*^−/−^ mice on days 50 (postnatal control) and 500 (pre-onset) were used in the analysis. The tumor site (DLBCL) was not included in the evaluation because it was assumed to exhibit significant changes in gene expression. The liver samples from 500-day-old female *DASPO*^*−/−*^ mice did not show grossly apparent tumor findings.

### Histopathology

The organs were fixed in 10% neutral buffered formalin and embedded in paraffin. At least five sections were prepared for histologic observation (hematoxylin and eosin staining) and immunohistochemistry. The deparaffinized and rehydrated sections prepared for immunohistochemistry were microwaved in 10 mM citrate buffer for 20 min in a microwave oven. After blocking endogenous peroxidase, these sections were incubated overnight at 4 °C with antibodies against CD3 (rabbit monoclonal, clone SP7, diluted 1:150; Abcam), CD20 (rabbit monoclonal, clone SP32, diluted 1:100; Abcam), CD10 (rabbit monoclonal, clone EPR22867-118, diluted 1:500; Abcam), or CD5 (rabbit polyclonal, diluted 1:250; Invitrogen), and then at room temperature for 1 h with Histofine® Simple Stain™ Mouse MAX PO® (Nichirei, Tokyo, Japan). After benzidine reaction, the sections were lightly counterstained with hematoxylin. As a negative control, isotype-matched immunoglobulin normal rabbit IgG (AB-105-C; R&D systems) was used as the primary antibody.

### RNA sequencing

Total RNA was extracted from the liver samples of 50- and 500-day-old *DASPO*^*−/−*^ and B6 mice (n = 4 per group) using the NucleoSpin RNA kit (Macherey–Nagel, Düren, Germany). The concentration and quality of the extracted RNA were determined using TapeStation and BioAnalyzer, respectively (Agilent Technologies, Santa Clara, CA, USA). cDNA libraries were generated from *DASPO*^*−/−*^ and B6 mouse liver RNA using the SMART-Seq v4 Ultra Low Input RNA Kit for Sequencing (Takara Bio, Japan) and the Nextera XT DNA Library Prep Kit (Illumina, San Diego, CA, USA) for RNA sequencing (RNA-Seq). RNA-Seq was performed on NovaSeq 6000 (Illumina) in the 2′ 150-bp paired-end mode, providing an average of 69 million reads for each sample. The RNA-Seq reads were mapped to the mouse genome (GRCm39), and gene-level expression was quantified (per gene read counts) using the DRAGEN Bio-IT Platform (Illumina). Differential gene expression analysis in all pairwise comparisons was performed with the read count dataset using the edgeR package (Robinson et al. 2010) in the R environment (version 4.2.0) (R Foundation for Statistical Computing, Vienna, Austria). Differentially expressed genes were determined based on Bonferroni-corrected *P* values and fold-changes in expression between groups. Gene Ontology (GO; biological process) term enrichment analysis of *DASPO*^*−/−*^-related genes was performed using the R package clusterProfiler.

### DASPO expression analysis in patients with DLBCL

Gene Expression Profiling Interactive Analysis 2 (GEPIA2) (Tang et al. 2019) (http://gepia2.cancer-pku.cn/#index) was used to analyze the relationship between DASPO expression and survival in patients with DLBCL.

### Statistical analysis

**Fig. 5:**
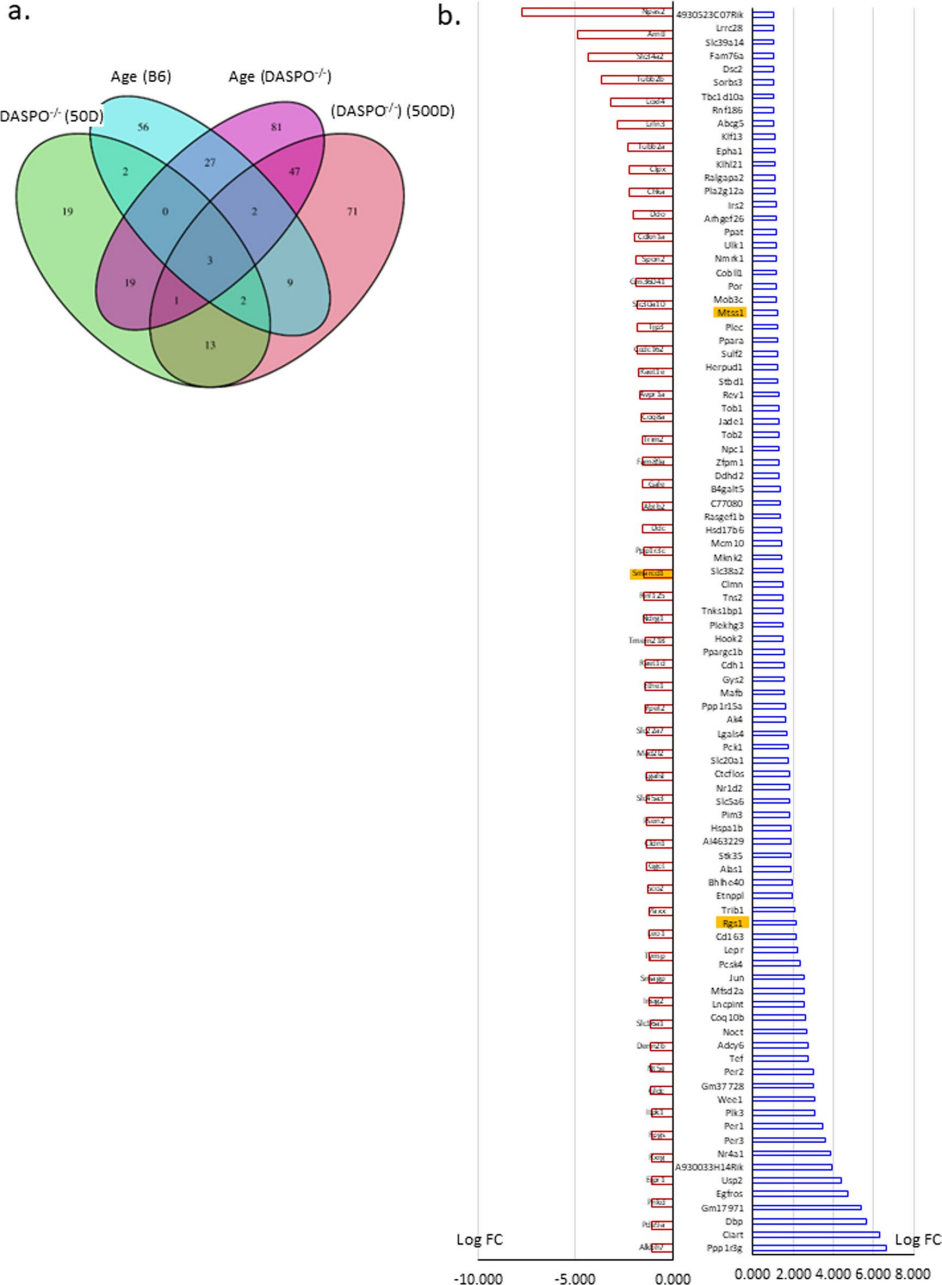

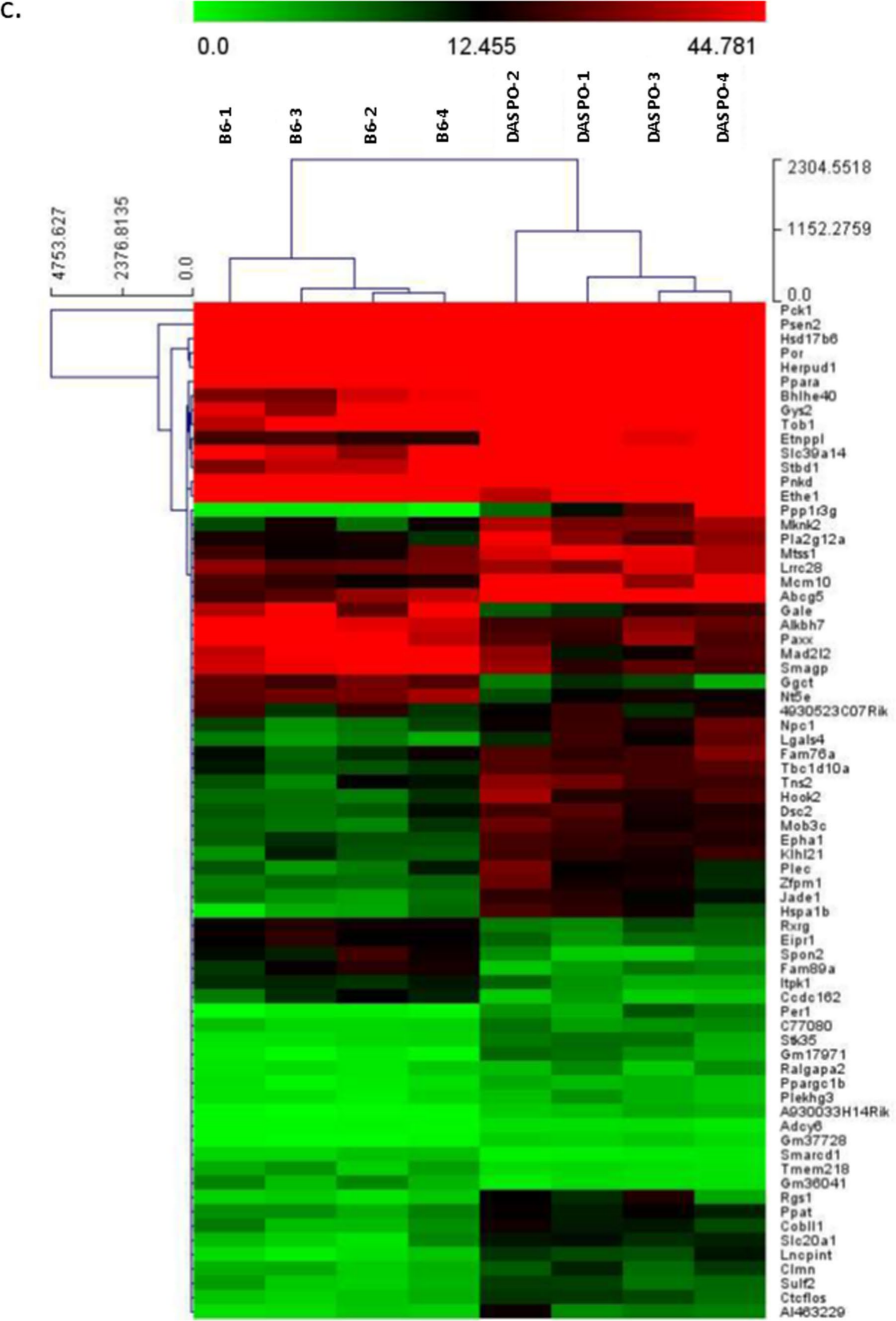

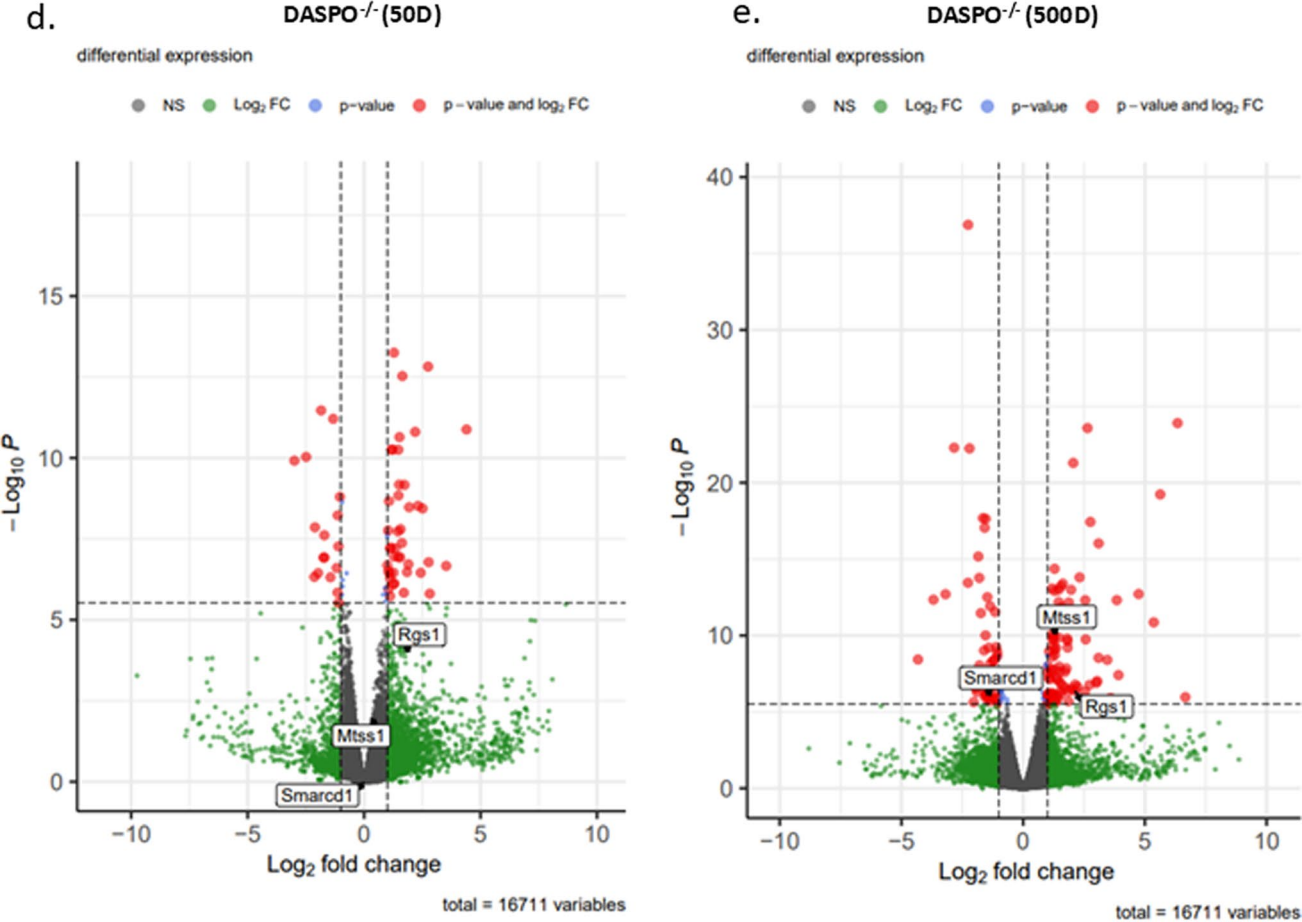
RNA-Seq analysis of the liver tissues from female *DASPO*^*−/−*^ mice. **a** Venn diagram. Age (B6): Number of genes whose expression was altered in 500-day-old female B6 mice compared with that in 50-day-old female B6 mice. Age (*DASPO*^*−/−*^): Number of genes whose expression was altered in 500-day female *DASP*^*−/−*^ mice compared with that in 50-day female *DASP*^*−/−*^ mice. *DASPO*^*−/−*^ (50D): Number of genes whose expression was altered in 50-day-old female *DASPO*^*−/−*^ mice compared with that in 50-day-old female B6 mice. *DASPO*^*−/−*^ (500D): Number of genes whose expression was altered in 500-day-old female *DASPO*^*−/−*^ mice compared with that in 500-day-old female B6 mice. **b** Expression levels. **c** Heatmap of 71 genes. Yellow highlights indicate DLBCL-related genes. **d:** Genes with altered expression with Bonferroni-corrected *P* < 0.05 are shown in red and those with expression ratio > twofold (logFC > 1 or logFC <  − 1) are shown in green for comparison between 50-day-old female *DASPO *^*−/−*^ mice and 50-day-old female B6 mice. **e:** Genes with altered expression with Bonferroni-corrected *P* < 0.05 are shown in red and those with expression ratio > twofold (logFC > 1 or logFC <  − 1) are shown in green for comparison between 500-day-old female *DASPO*^*−/−*^ mice and 500-day-old female B6 mice. DASPO, D-aspartate oxidase

Student’s *t*-test and Mann–Whitney U test were used to compare two groups (Fig. 1). A log-rank test was performed for survival time analysis (Fig. 2). The Bonferroni method was used to compare gene expression (Fig. 5 and Online Resources 3–5). Statistics version 23 software (IBM, Tokyo, Japan) and R (https://www.r-project.org/) were used for statistical analyses. Results were considered significant at *P* < 0.05.

The animal experiments have been described according to the ARRIVE 2.0 guidelines (The ARRIVE Guidelines 2.0 2024).

## Results

### Plasma amino acid profiles of D-AA-related metabolic enzyme-knockout mice

The multidimensional data from the plasma D-amino acid analysis results were plotted in a low-dimensional plot using the principal coordinate analysis (PCoA) to visualize diversity and similarity. For example, in Fig. 1a (left), the x-axis of the loading data demonstrates the difference between B6 and *DAAO*^−/−^ mice. Deviation from the 0.0 value signifies a higher discrimination ability. The y-axis indicates the influence of other factors, such as sex. Figure 1a (right) shows the outcomes of distinguishing *DAAO*^−/−^ mice (red dots) from B6 mice (blue dots) using these amino acids. The results of the ROC analysis, which showed the greatest difference between D-AA gene-deficient and B6 mice, are also shown.

The PCoA results showed that *DAAO*^*G181R*^ mice had different plasma D-AAs profiles compared to B6 mice, and the ROC analysis showed that plasma levels of D-Proline and D-Serine were significantly higher in *DAAO*^−/−^ than in B6 mice (Fig. 1a). PCoA results showed a different trend in the D-AA profiles of male and female *DAAO*^*G181R*^ mice. However, no sex differences were observed in the levels of DAAO substrates D-alanine and D-serine (Fig. 1b).

The PCoA results confirmed that *DASPO*^−/−^ mice had different plasma D-AA profiles compared to B6 mice. The ROC analysis showed that the plasma level of D-aspartate, a substrate of DASPO, was significantly higher in *DAAO*^−/−^ mice than in B6 mice (Fig. 1c). PCoA results showed a different trend in the D-AA profiles of male and female *DASPO*^−/−^ mice. However, no sex differences were observed in the levels of DASPO substrates D-glutamate and D-aspartate (Fig. 1d).

The PCoA results confirmed that *SR*^−/−^ mice had different plasma D-AA profiles compared to B6 mice. The ROC analysis showed that the level of D-serine, a substrate of SR, was significantly lower in *SR*^−/−^ mice than in B6 mice (Fig. 1e). PCoA results showed a different trend in the D-AA profiles of male and female *SR*^−/−^ mice. However, no significant difference in D-serine level was observed between male and female mice (Fig. 1f).

These results indicate that mice deficient in the DAA-related metabolic enzyme gene have altered plasma D-AA profiles.

### Lifespan of D-AA-related metabolic enzyme-knockout mice

The lifespan of female *DASPO*^*−/−*^ mice was the shortest among all groups of mice (log-rank, *P* = 0.007) (Fig. 2a). Next, only female mice were evaluated to correct for the effects of sex differences. Even under these conditions, the lifespan of female *DASPO*^*−/−*^ mice was shorter than that of the other group mice (log-rank, *P* = 0.045) (Fig. 2b).

### Changes in the macroscopic findings of organs in D-AA-related metabolic enzyme-knockout mice

Macroscopic findings of organs were evaluated to determine the cause of death of female *DASPO*^*−/−*^ mice. Female B6 mice showed no masses in their organs (Fig. 3a). However, in female *DASPO *^*−/−*^ mice (Fig. 3a, b), mice expressing a natural variant that inactivates DAAO (*DAAO*^*G181R*^) (Fig. 3a and Online Resource 1a), and *SR*^*−/−*^ mice (Fig. 3a and Online Resource 1b), masses were observed in the liver, spleen, and small intestine. These masses were especially frequent in the liver of female *DASPO*^*−/−*^ mice (Fig. 3a).

Male B6 mice showed masses in the liver, spleen, and mesenteric lymph nodes. Male *DASPO*^*−/−*^ (Online Resource 2a, b), *DAAO*^*G181R*^ (Online Resource 2a, c), and *SR*^*−/−*^ mice (Online Resource 2a, d) also showed masses in the liver, spleen, small intestine, and mesenteric lymph nodes. Although no masses were observed in the kidneys, blisters were noted in B6, *DAAO*^*G181R*^, and *SR*^*−/−*^ mice (Online Resource 2c).

### Pathological findings of each organ in female *DASPO*^−/−^ mice

To identify the cause of mass development in female *DASPO*^*−/−*^ mice (Fig. 4), we analyzed the pathological findings of the organs. Medium or large atypical lymphocytes were distributed in the thoracoabdominal organs. The liver, showing a suspected relation to death, showed atypical lymphocytes diffusely infiltrating the sinusoidal space with scattered mass-forming cells (Fig. 4a, b). An abnormal localized dilatation was macroscopically recognized in the small intestine. In the distal neighborhood, a microscopic examination revealed cellular thickening of the intestinal wall and marked infiltration and mass formation by atypical lymphocytes from the submucosal to subserosal layers without mucosal involvement (Fig. 4c, d). In the myocardium, atypical lymphocytes were scattered in the atrial septum and focally accumulated in the impulse-conducting system (Fig. 4e). The kidney showed dilatation of the proximal tubule and mild lymphocytic infiltration in the interstitial space. The spleen had scattered multinucleated megakaryocytes, indicating extramedullary hematopoiesis (Fig. 4f). The immunohistochemical analyses revealed that these proliferating lymphocytes were mostly CD20-positive B lymphocytes and that CD3-positive T lymphocytes were sparse (Fig. 4g, h). Based on the pathological findings, DLBCL or follicular lymphoma was considered a differential diagnosis; however, DLBCL was confirmed by the abundant Ki67-positive cells and a lack of definite follicular formation. The mass formed by these lymphoma cells was limited to the liver and small intestine and was not found in any systemic lymph nodes.

### RNA-Seq of liver samples from female *DASPO*^−/−^ mice

The results are presented as a Venn diagram based on Bonferroni-corrected *P* < 0.05 and expression ratio > twofold (logFC > 1 or logFC < − 1) (Fig. 5a). A comparison of 50- and 500-day-old female B6 mice revealed that the expression of 101/16 711 genes was altered by aging (Fig. 5a: Age [B6]; Online Resource 3). A similar comparison of 50- and 500-day-old female *DASPO*^*−/−*^ mice indicated that the expression of 180/16 711 genes was altered by aging (Fig. 5a: Age [*DASPO*^*−/−*^]; Online Resource 4). A comparison of 50-day-old female B6 mice with 50-day-old female *DASPO*^*−/−*^ mice revealed that the expression of 59/16 711 genes was altered at baseline (Fig. 5a: *DASPO*^*−/−*^ [50D]; Online Resource 5). Excluding the genes whose expression was altered with aging and at baseline, the expression of 71 genes was found to be altered in the comparison between 500-day-old female B6 and *DASPO*^*−/−*^ mice (Fig. 5a: *DASPO*^*−/−*^ (500D), B). Multiple Experiment Viewer (MeV; https://mev.tm4.org/#/about) was used to analyze the clustering of the 71 *DASPO*^*−/−*^-related genes. This analysis also confirmed that genes in B6 and *DASPO*^*−/−*^ mice can be clustered into two major groups (Fig. 5c). Among the 71 *DASPO*^*−/−*^-related genes, *RGS 1*, *MTSS1*, and *SMARCD 1* are known DLBCL-related genes (Fig. 5b, d, e). No tumors were grossly and histologically confirmed in the livers of the 50- and 500-day-old mice sampled. Therefore, this change was not tumor-induced but was caused by a gene affected by the genetic background of *DASPO*^*−/−*^ mice.

The GO analysis to predict the biological processes of the 71 *DASPO*^*−/−*^-related genes revealed three GO terms: generation of precursor metabolites and energy, cellular carbohydrate metabolic process, and carbohydrate biosynthetic process (Online Resource 6a, b).

### Kaplan–Meier curve of D-AA-related enzyme gene expression levels in patients with DLBCL

**Fig. 6:**
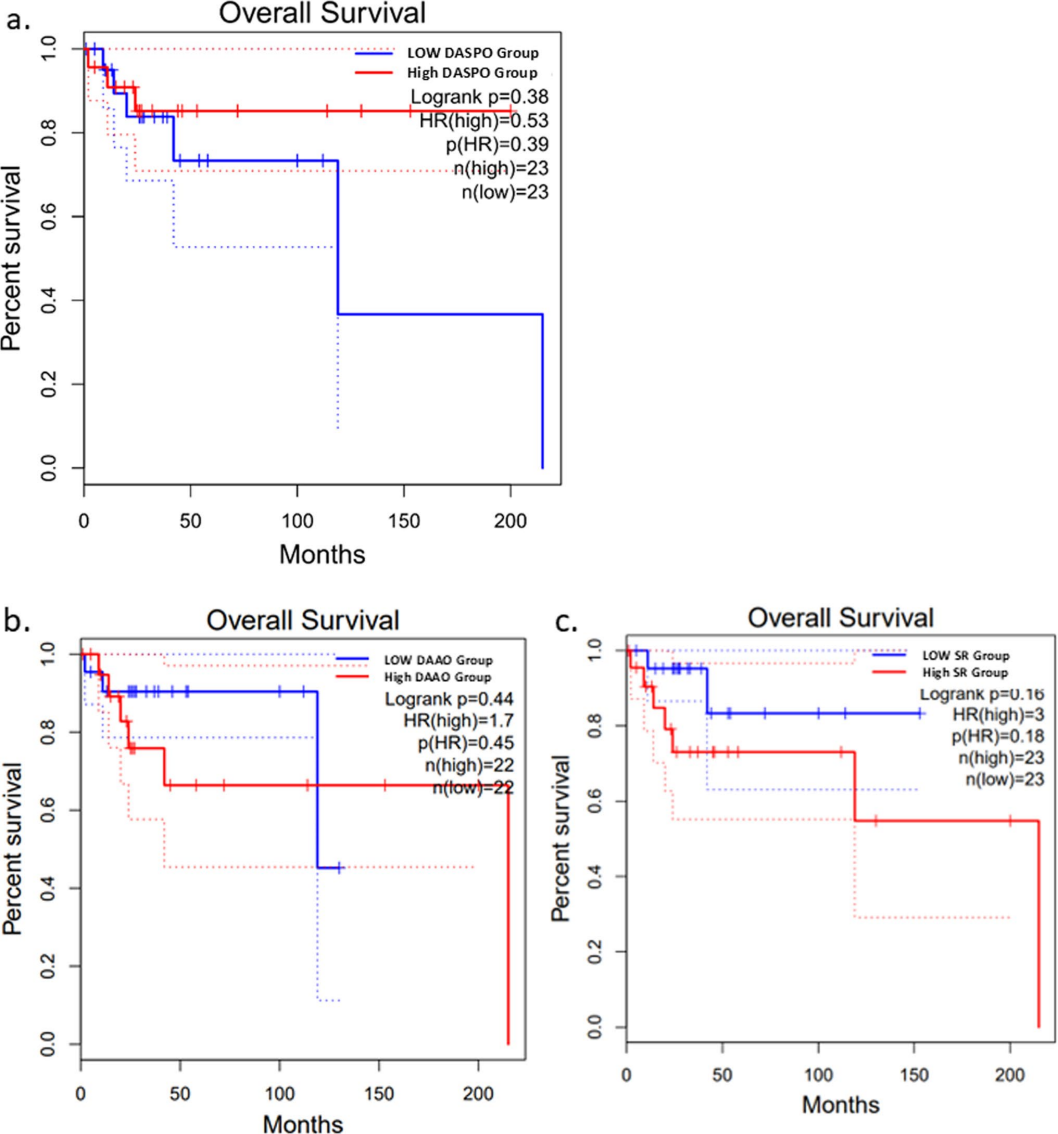
Kaplan–Meier curve of D-AA-related enzyme gene expression levels generated using GEPIA2. Survival analysis based on human **a** *DASPO*, **b** *DAAO*, and **c** *SR* gene expression levels. Adapted from GEPIA2 (http://gepia2.cancer-pku.cn). DASPO, D-aspartate oxidase; DAAO, D-amino acid oxidase; SR, serine racemase

The low-DASPO group showed a trend toward a shorter survival period than the high-DASPO group (Fig. 6a). In contrast, the low- and high-DAAO groups showed no changes in survival (Fig. 6b). The low- and high-SR groups also showed no changes in survival (Fig. 6c). These results in humans were consistent with those in mice.

## Discussion

In the present study, after long-term rearing, mice deficient in D-amino acid-related metabolic enzymes showed tumor development. Female *DASPO*^*−/−*^ mice exhibited a higher frequency of mass formation and shorter lifespan, and the masses were pathologically diagnosed as DLBCL. The expression of 71 genes was altered in *DASPO*^*−/−*^ mice. Additionally, human patients with DLBCL showing low DASPO expression presented shorter survival period. These findings suggest that congenital dysregulation of DASPO may contribute to DLBCL development.

In animals, DASPO is expressed in tissues throughout the body and is particularly active in the brain, lung, liver, spleen, and kidney (Van Veldhoven et al. 1991). Similarly, in humans, DASPO expression has been confirmed in all organs (Online Resource 7) (Uhlén et al. 2015). In our human study, patients with DLBCL with lower *DASPO* expression tended to have shorter survival rates than those with higher *DASPO* expression (Fig. 6). Interestingly, DASPO expression is higher in female individuals than in male individuals (Kera et al. 1993; Nagasaki 1994). In the present study, the lifespan of female *DASPO*^*−/−*^ mice was shorter than that of male *DASPO*^*−/−*^ mice, suggesting that female mice may be more affected by DASPO deficiency than male mice. This interpretation is consistent with the findings of Kera et al. (1993) and Nagasaki (1994).

In addition, D-Asp and D-Glu, substrates of DASPO, have been reported to be associated with the development of cancer (Du et al. 2019; Han et al. 2015, 2018). *DASPO*^*−/−*^ mice had higher plasma D-Asp levels compared to B6 mice. On the other hands, there was no difference for D-Glu (Fig. 1). Therefore, it was thought that D-Asp might affect carcinogenesis in this study. An association between D-Asp levels and cancer has been reported. In human MCF-7 breast cancer cells, the D-Asp level is higher than that in non-neoplastic MCF-10A breast epithelial cells (Du et al. 2019). At this time, however, there are many unknowns regarding the relationship between D-Asp and DLBCL and the influence of gender differences. Overall, the relationship between DSPAO deficiency and DLBCL development needs further research.

In the present study, histopathology indicated that the tumors developed in female *DASPO*^*−/*−^ mice were DLBCL. Then, we assumed the underlying metabolic change in tissue cell, especially micro environments of liver, may induces prominent tumor growth. Therefore, we examined alterations in the expression of genes associated with DLBCL development in these mice. RNA-Seq of the liver, which had the highest tumor frequency, showed that the expression of 71 genes was specifically altered in *DASPO*^*−/−*^ mice compared with that in B6 mice; these genes included *RGS 1*, *MTSS1*, and *SMARCD 1*, which are associated with DLBCL development. High *RGS 1* expression is associated with poor overall survival in patients with DLBCL (Carreras et al. 2017), consistent with our results. *RGS 1* upregulation is reportedly associated with shorter survival period in patients with breast and lung cancers (Huang et al. 2021) and those with DLBCL. The association between *RGS 1* and cancer development reportedly involves its upregulated expression via type II interferon-signal transducer and activator of transcription 1 signaling and impaired trafficking of circulating T cells to tumors via inhibition of calcium influx and suppressed activation of the kinases ERK and AKT. Furthermore, *RGS 1* is associated with CD8+ T-cell exhaustion (Bai et al. 2021). *MTSS1* expression was upregulated in mice in the present study. The relationship between *MTSS1* expression and DLBCL development has been reported (Xu and Xu 2018; Du et al. 2022). Decreased *MTSS1* expression promotes the proliferation, invasion, and metastasis of DLBCL OCI-LY10 cells (Xu and Xu 2018). In contrast, patients with high levels of *MTSS1* transcripts have a favorable prognosis compared to those with reduced or no expression (Du et al. 2022). In addition to these three genes, we identified 68 genes whose expression was altered, and they may be novel DLBCL-related genes. Some of these genes were predominantly related to the generation of precursor metabolites and energy, cellular carbohydrate metabolic process, and carbohydrate biosynthetic process as indicated by the GO analysis. The association between these genes and DLBCL development needs to be investigated to elucidate the novel pathogenesis of DLBCL.

In the present study, mice deficient in D-AA-related enzymes, DAAO and SR, showed no changes in lifespan but showed the formation of masses. DAAO has attracted attention as a candidate for a new enzyme prodrug therapy in cancer treatment. Previous studies have confirmed that hydrogen peroxide generated by DAAO exerts cytotoxicity on tumor cells (EI Sayed et al. 2012). In particular, efforts are being made to enhance the tumor specificity of the enzyme and suppress adverse effects via modification using protein engineering and nanotechnology (Rosini et al. 2020, 2021, 2022). It has also been reported that DAAO expression is associated with B cell regulation, suggesting that metabolic changes in D-amino acids and DLBCL may be related (Suzuki et al. 2021). SR is reportedly associated with cancer development (Miyoshi et al. 2012a, b; Ohshima et al. 2020). SR activation supports the growth of colorectal cancer cells, whereas SR inhibition inhibits cancer growth (Ohshima et al. 2020). Thus, although there are only a few reports, D-AA-related metabolic enzymes and cancer may be closely related. Further elucidation of the detailed mechanism is needed.

This study has some limitations. Although we evaluated underlying metabolic changes in liver, in which prominent tumors are identified, gene analysis of B cell is not performed. Therefore, the association between DASPO deficiency and development of DLBCL remains unclear. In the future, it is necessary to focus on B cells and investigate the mechanism of DLBCL development in association with DASPO deficiency. As D-AA-related metabolic enzyme-deficient mice showed diverse phenotypes, organ and histological abnormalities other than those affected by DLBCL may be influenced by D-AA-related enzyme deficiency. Furthermore, brain function and nervous system activity were not evaluated. Organs such as the eye, thyroid, testis, and ovary were also not examined, warranting further research.

Overall, this study showed that *DASPO*^*−/−*^ mice have a shorter lifespan because of changes in the expression of 71 genes and development of DLBCL. Patients with DLBCL who showed low *DASPO* expression also showed a trend of poor long-term prognosis compared to those who showed high *DASPO* expression. Furthermore, DAAO, DASPO, and SR deficiencies were found to induce tumor-like mass development in mice. These results elucidate the importance of *DASPO* as novel biomarkers and therapeutic targets in cancer.

## Data availability statement

The RNA-Seq data (FASTQ reads) generated have been deposited at the DNA Data Bank of Japan (DDBJ) under DDBJ Sequence Read Archive (DRA) accession number DRA014804.

## Supplementary Information

Below is the link to the electronic supplementary material.

Supplementary file1 (DOCX 3135 KB)

## Declarations

### Research involving animals

All animals were maintained and used according to Kanazawa University guidelines, and experiments were conducted upon approval from Kanazawa University (approval no. AP-204160).
