# Supplementary material for: Effect of D-amino acid metabolic enzyme deficiency on cancer development—diffuse large B-cell lymphoma onset and gene expression analyses in DASPO-knockout mice
Source: Amino Acids. 2024 Dec 24;57(1):4. doi: 10.1007/s00726-024-03426-1 (PMC11668858; doi:10.1007/s00726-024-03426-1)
Supplement: Supplementary file 1 — Supplementary file1 (DOCX 3135 KB) [file 726_2024_3426_MOESM1_ESM.docx]

**Online Resources**

**Amino Acids**

**Effect of D-Amino Acid Metabolic Enzyme Deficiency on Cancer Development—Diffuse Large B-Cell Lymphoma Onset and Gene Expression Analyses in DASPO-Knockout Mice**

Yusuke Nakade, Yasunori Iwata, Kenichi Harada, Yasuharu Sato, Masashi Mita, Kenji Hamase, Ryuichi Konno, Mayo Hayashi, Taku Kobayashi, Yuta Yamamura, Tadashi Toyama, Atsushi Tajima, and Takashi Wada ^*^

***Correspondence:** Takashi Wada; twada@staff.kanazawa-u.ac.jp


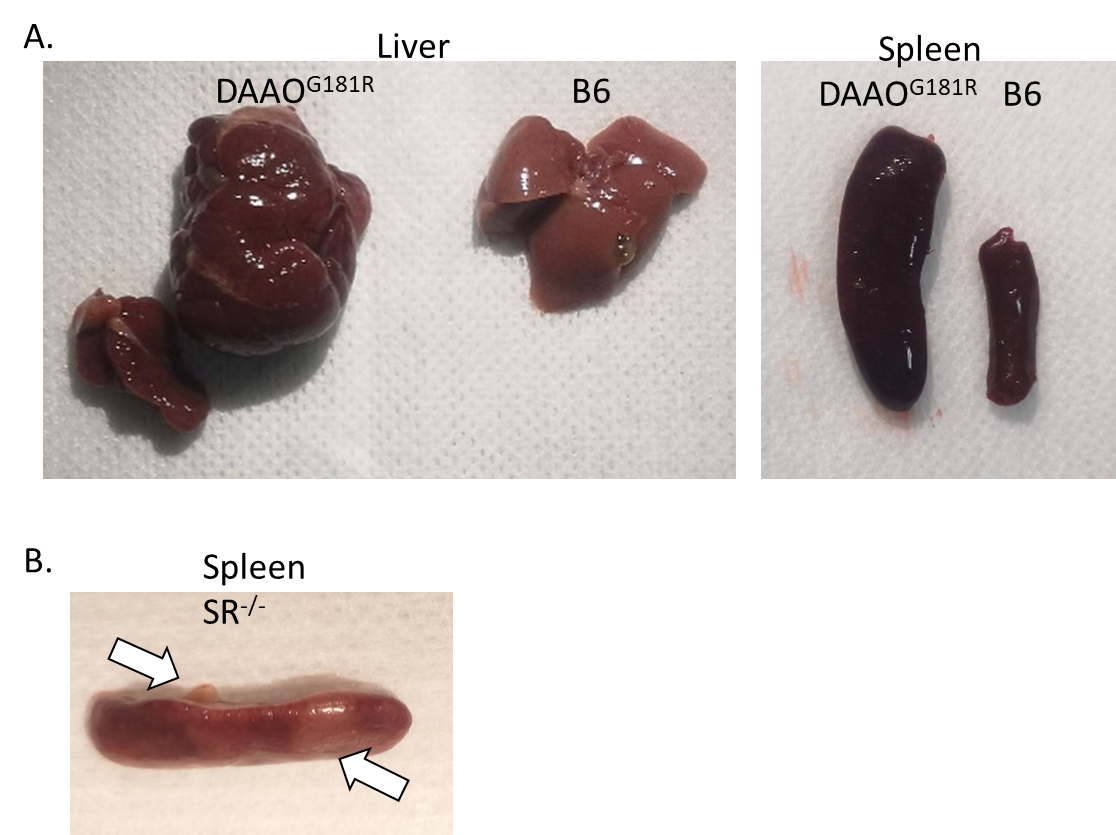


Online Resource 1. Macroscopic findings of organs in female *DAAO^G181R^* and *SR^-/-^* mice.

Macroscopic findings of organs were evaluated to determine the cause of death of female *DAAO^G181R^* and *SR^-/-^* mice. The mice were sacrificed at approximately 900 days of age. Macroscopic findings of organs in female *DAAO^G181R^* **(a**) and *SR^-/-^* (**b**) mice.

DAAO, D-amino acid oxidase; SR, serine racemase


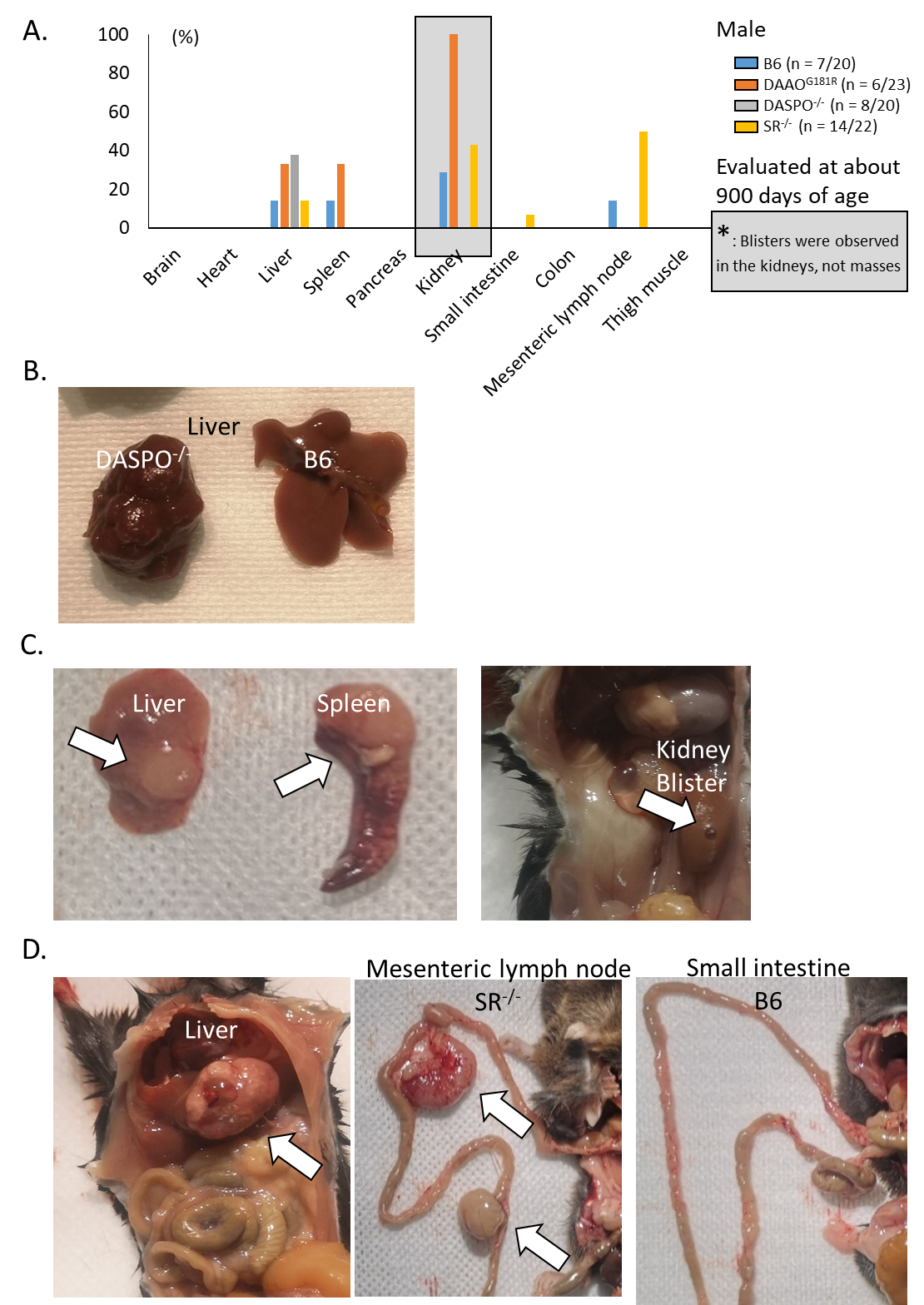


Online Resource 2. Macroscopic findings of organs in male mice.

Mice were sacrificed at approximately 900 days of age. (**a**) Frequency of mass formation in male B6 and D-AA-related metabolic enzyme knockout (*DAAO^G181R^*, *DASPO ^-/-^*, and *SR^-/-^*) mice. Macroscopic findings of organs in male *DASPO ^-/-^* (**b**), *DAAO^G181R^* **(c**), and *SR^-/-^* mice (**d**).

DAAO, D-amino acid oxidase; DASPO, D-aspartate oxidase; SR, serine racemase


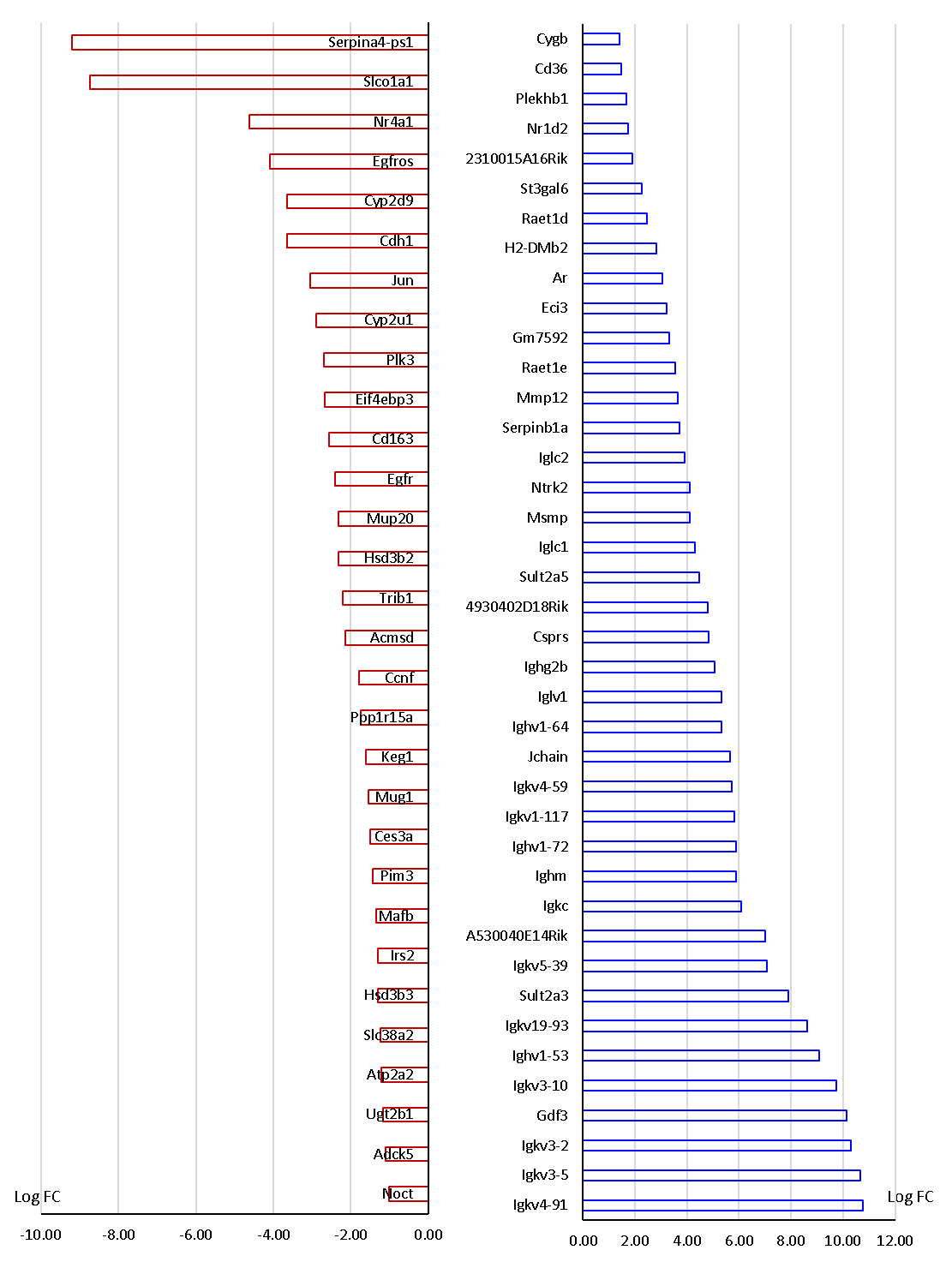


Online Resource 3. Group of genes related to aging determined using RNA-Seq analysis of the liver tissues from female B6 mice.

Expression of genes changed in 500-day-old female B6 mice when compared with that in 50-day-old female B6 mice.

DASPO, D-aspartate oxidase


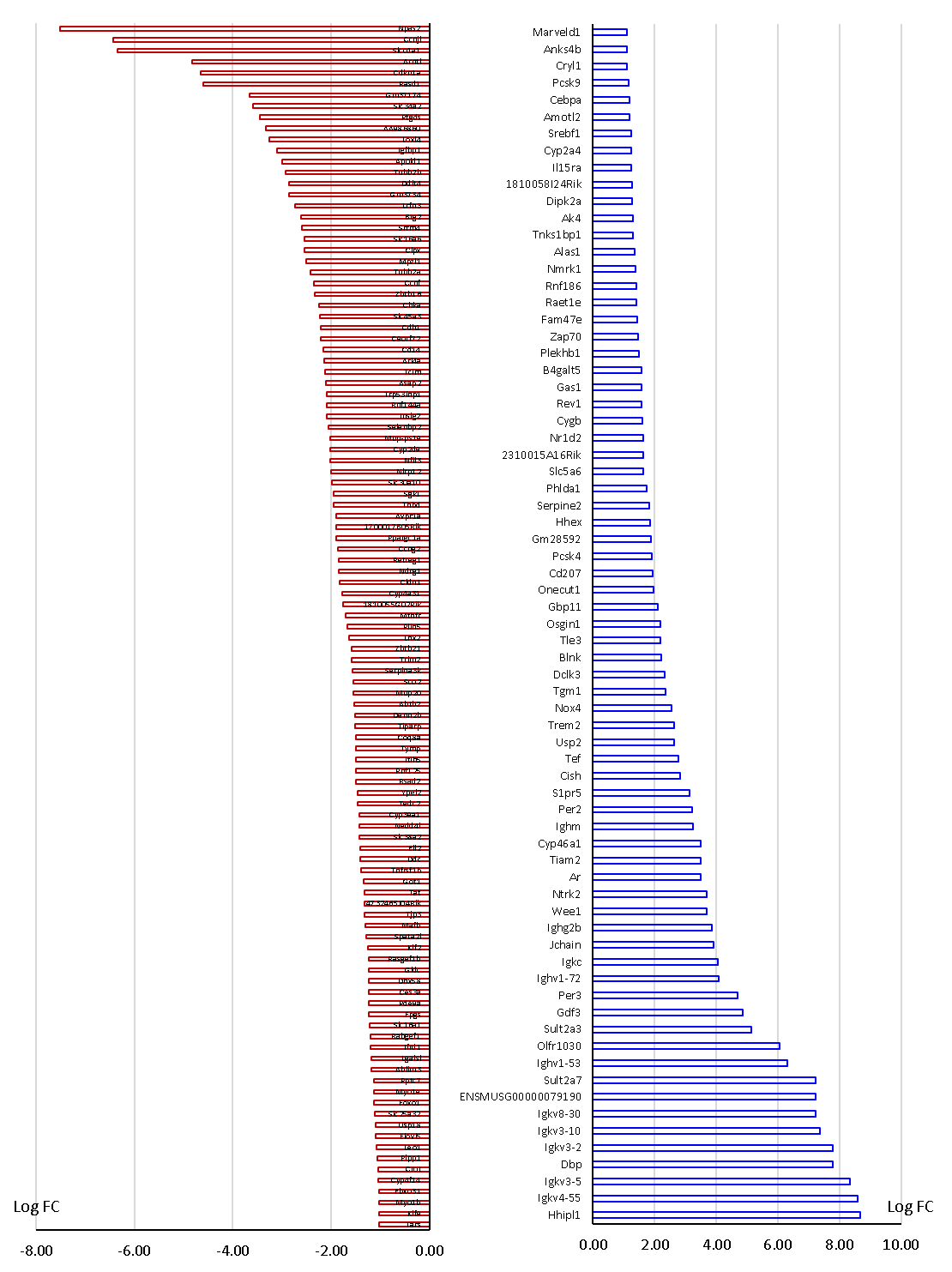


**Online Resource** **4.** **Group of genes related to aging determined using RNA-Seq analysis of the liver tissues from female** *DASPO* **^-/-^ mice.**

The expression of genes changed in 500-day-old female *DASPO ^-/-^* mice when compared with that in 50-day-old female *DASPO ^-/-^* mice.

DASPO, D-aspartate oxidase


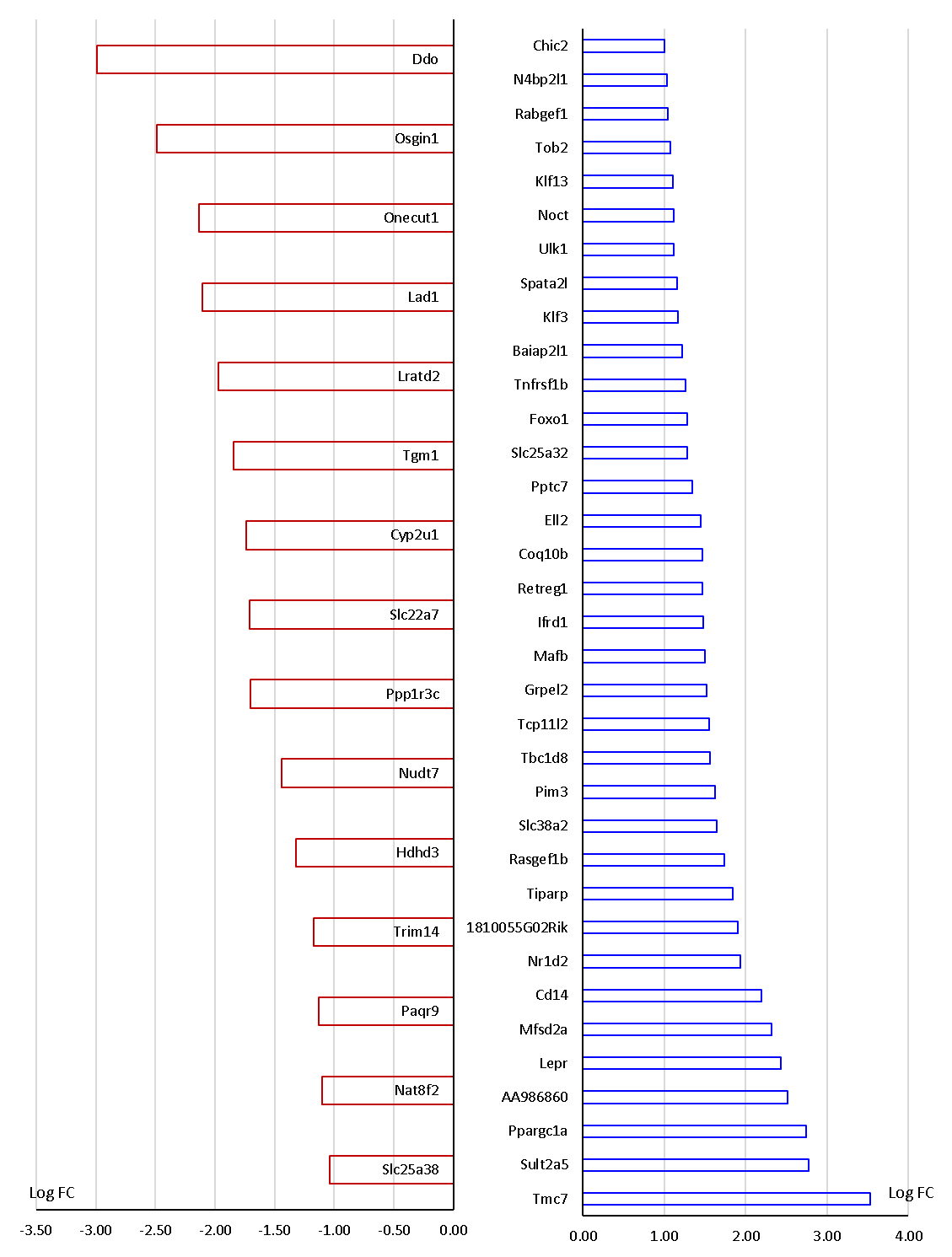


**Online Resource** **5.** **Genetic differences at baseline between female B6 and female** *DASPO* ***^-/-^* mice.**

The expression of genes changed in 50-day-old female B6 mice when compared with that in 50-day-old female *DASPO ^-/-^* mice.

DASPO, D-aspartate oxidase


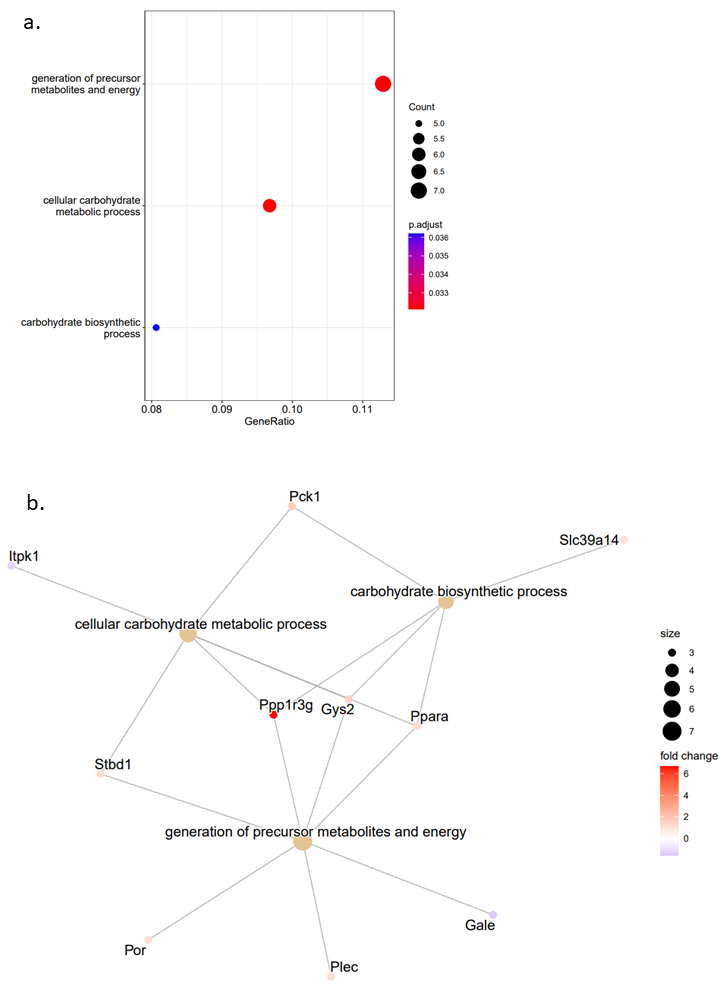


**Online Resource 6.** Gene Ontology analysis of 71 *DASPO ^-/-^*-related genes. **a:** Enriched biological processes (dots). **b:** Enriched biological processes (network).


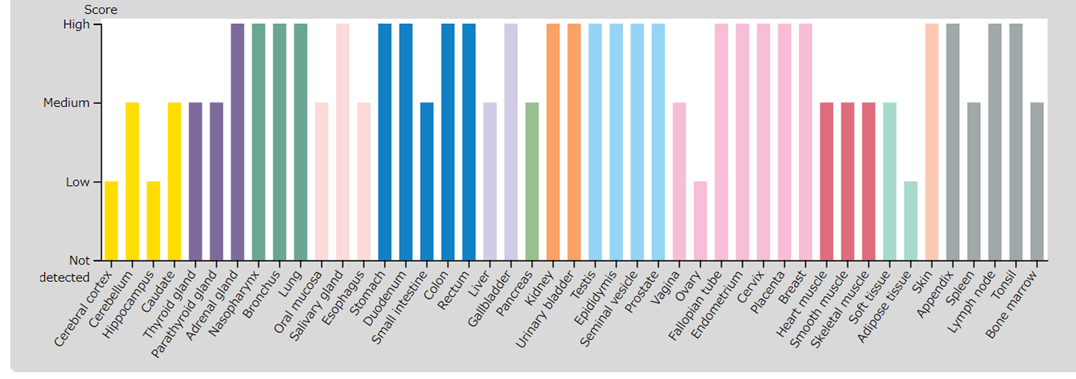


**Online Resource 7.** **DASPO protein expression scores in human tissues.**
